# *In vitro* susceptibility of *Stenotrophomonas maltophilia* and other carbapenem non-susceptible Gram-negative rods in the Pacific Northwest to cefiderocol and aztreonam-avibactam

**DOI:** 10.1093/jacamr/dlag105

**Published:** 2026-06-08

**Authors:** Andrew Bryan, Megan Shields-Pruiett, Heather Berger, Ferric C Fang, Kelsi Penewit, Adam Waalkes, Elizabeth A Holmes, Stephen J Salipante, Brian J Werth

**Affiliations:** Department of Laboratory Medicine and Pathology, Mayo Clinic Arizona, Phoenix, USA; Laboratory Services, Samaritan Health Services, Lebanon, OR, USA; Department of Laboratory Medicine and Pathology, University of Washington, Seattle, WA, USA; Department of Laboratory Medicine and Pathology, University of Washington, Seattle, WA, USA; Department of Microbiology, University of Washington, Seattle, WA, USA; Department of Laboratory Medicine and Pathology, University of Washington, Seattle, WA, USA; Department of Laboratory Medicine and Pathology, University of Washington, Seattle, WA, USA; Department of Laboratory Medicine and Pathology, University of Washington, Seattle, WA, USA; Department of Laboratory Medicine and Pathology, University of Washington, Seattle, WA, USA; Department of Pharmacy, University of Washington, Seattle, WA, USA

## Abstract

**Objectives:**

Carbapenem resistance is a growing public health crisis with regional variability in incidence. We characterized the susceptibility of carbapenem-resistant Gram-negative rods (CR-GNRs) from the Pacific Northwest to five reserve beta-lactam agents including cefiderocol and aztreonam-avibactam.

**Methods:**

A panel of 162 CR-GNRs from unique patients were tested against cefiderocol, aztreonam-avibactam, ceftazidime-avibactam, meropenem-vaborbactam and ceftolozane-tazobactam, with a subset of isolates derived from patients with bacteraemia (*n* = 55). Whole-genome sequencing (WGS) was performed on representative *Stenotrophomonas maltophilia* isolates displaying a range of cefiderocol MICs (*n* = 9).

**Results:**

Among all isolates, the MIC_50_ and MIC_90_ to cefiderocol was 0.25 and 4 mg/L, respectively, compared with 4 and >64 mg/L for aztreonam-avibactam. Overall, 98.3% of *S. maltophilia* (*n* = 60), 91.2% of Enterobacterales (*n* = 34) and 88.5% of *Pseudomonas aeruginosa* (*n* = 26) isolates were susceptible to cefiderocol, while 78.6% of other non-fermenters had MICs ≤4 mg/L. Aztreonam-avibactam and cefiderocol exhibited similar activity against Enterobacterales and *S. maltophilia*, but aztreonam-avibactam was less active against other organisms. WGS of *S. maltophilia* isolates with elevated cefiderocol MICs identified disruptive mutations in PBP-encoding genes (*mrcB/*PBP1b and *pbpC/*PBP1c) that were not observed in highly susceptible isolates. The bacteraemia cohort had many comorbidities and a 29% 30 day all-cause mortality. In this cohort, cefiderocol demonstrated the highest *in vitro* activity as extrapolated from MICs, followed by ceftazidime-avibactam, aztreonam-avibactam, meropenem-vaborbactam and ceftolozane-tazobactam, respectively.

**Conclusions:**

Cefiderocol exhibited the greatest *in vitro* activity against various CR-GNRs species in a Pacific Northwest population enriched for severely ill patients. Susceptibility to other agents was more variable among tested organisms.

## Introduction

Carbapenem-resistant Gram-negative rods (CR-GNRs) represent a growing global challenge, with rates and mechanisms varying geographically and by species.^1^ While newer beta-lactam/beta-lactamase inhibitor combinations have increased therapeutic options, metallo-beta-lactamases (MBLs), including NDM, VIM, IMP and the L1 beta-lactamase of *Stenotrophomonas maltophilia* remain particularly challenging. Susceptibility data are increasingly available for carbapenem-resistant Enterobacterales but are currently limited for many non-fermenters, particularly for the newer agents.^2–4^

Cefiderocol and aztreonam-avibactam are active against many CR-GNRs, including MBL producers, although resistance has been described.^5–7^ For immunocompromised patients and those with cystic fibrosis, who have an increased risk of infections with non-fermenting organisms, understanding the *in vitro* activity of reserve beta-lactam agents is critical for guiding empiric therapy when CR-GNRs are suspected.^8^

Given regional variability in resistance, we assessed susceptibility of CR-GNRs to five reserve beta-lactams—including cefiderocol and aztreonam-avibactam—in the North American Pacific Northwest. Additionally, we described clinical outcomes for patients with CR-GNR bacteraemia and performed whole-genome sequencing (WGS) to explore genetic traits associated with cefiderocol resistance in *S. maltophilia*.

## Methods

### Selection of isolates and patients

We identified 162 unique GNR isolates based on clinical testing from 161 patients at the University of Washington Medical Center and Harborview Medical Center from 5/2008–6/2021 (Table S1, available as Supplementary data at *JAC-AMR* Online). Carbapenem non-susceptible inclusion criteria included the following: (i) intrinsically resistant to meropenem/imipenem (e.g. *S. maltophilia*) or phenotypically tested non-susceptible to meropenem (applies to 154 isolates), (ii) imipenem non-susceptible and cefepime non-susceptible (applies to 1 *Achromobacter* and 1 *Pseudomonas*), (iii) ertapenem non-susceptible and positive by the carbapenem inactivation method (applies to 4 *Enterobacter* and 1 *Serratia*) or (iv) ertapenem non-susceptible and positive by the Cepheid Xpert Carba-R test for a carbapenemase (applies to 1 *E. cloacae* NDM). The collection of isolates was enriched for *S. maltophilia* and underrepresented non-fermenters, while a subset of bacteraemia isolates was chosen for clinical review without enrichment for particular organisms. Demographic information, comorbidities and 30 day survival were extracted by chart review. The study was approved by the University of Washington Institutional Review Board (STUDY00007292).

### Susceptibility testing

Cefiderocol MICs were measured by broth microdilution (BMD) using cation-adjusted iron-depleted Mueller–Hinton broth using supplied protocols and reading guidelines (Shionogi Inc. through International Health Management Associates; IHMA). Performance was assessed in 30 isolates with known reference broth dilution MICs provided by the sponsor via IHMA. Aztreonam-avibactam MICs were measured by agar dilution with performance assessed using CLSI QC ranges and inferred from isolates with known resistance.^9,10^ Ceftazidime-avibactam, ceftolozane-tazobactam and meropenem-vaborbactam MICs were measured by gradient strip diffusion (bioMerieux). All other MICs were performed and reported clinically prior to the initiation of this study (e.g. Table S3). Cefiderocol, ceftazidime-avibactam, ceftolozane-tazobactam and meropenem-vaborbactam MICs were interpreted per CLSI M100 35th Ed.^10^ Aztreonam-avibactam was interpreted per FDA breakpoints.^11^ For organism–agent combinations lacking established breakpoints, extrapolated susceptibility was interpreted using breakpoints from related species (Table 1).

**Table 1.** Minimum inhibitory concentration (MIC) distributions

	Agent	MIC_50_	MIC_90_	Range	Susceptible CLSI/FDA Bp^	Susceptible extrapolated#
*S. maltophilia* (*n* = 60)	FDC	0.06	0.125	≤0.03 to 2	98.3%	—
	ATM-AVI	2	4	≤0.03 to 8	—	95.0%
	C/T	16	>256	0.5 to >256	—	36.7%
	CZA	16	>256	0.5 to >256	—	46.7%
	MVB	>256	>256	1 to >256	—	3.3%
Enterobacterales (*n* = 34)	FDC	1	4	0.03 to 32	91.2%	—
	ATM-AVI	0.25	1	≤0.03 to 64	91.2%	—
	C/T	8	>256	0.25 to >256	35.3%	—
	CZA	1	>256	0.06 to >256	79.4%	—
	MVB	0.25	8	0.03 to >256	85.3%	—
*P. aeruginosa* (*n* = 26)	FDC	0.125	8	0.06 to >32	88.5%	—
	ATM-AVI	16	32	≤0.03 to >64	—	30.8%
	C/T	4	>256	0.5 to >256	50%	—
	CZA	8	>256	1 to >256	50%	—
	MVB	16	64	1 to >256	—	15.4%
Other non-fermenting	FDC	1	16	≤0.03 to >32	—	78.6%*
GNRs (*n* = 42)	ATM-AVI	>64	>64	2 to >64	—	2.4%
	C/T	4	>256	≤0.016 to >256	—	47.6%
	CZA	8	>256	1 to >256	—	50.0%
	MVB	32	>256	0.25 to >256	—	26.2%

MIC values reported in mg/L. Breakpoints applied: ^CLSI M100 35th Ed 2025 (FDC, C/T, CZA, MVA) or FDA (ATM-AVI). # ‘Extrapolated’ breakpoints applied: For CZA, C/T and FDC without formal interpretations, CLSI M100 35th Ed *P. aeruginosa* applied. For ATM-AVI without interpretations, FDA Enterobacterales breakpoints applied. For MVA, CLSI M100 35th Ed MEM applied for *P. aeruginosa*, *Acinetobacter*, *Stenotrophomonas*, other non-Enterobacterales or 33rd Ed MEM for *Burkholderia* spp. *Includes 4 *Acinetobacter* isolates that tested susceptible by CLSI M100 35th Ed breakpoints.

### WGS of *S. maltophilia*

WGS library preparation, sequencing and analysis was performed on five isolates of *S. maltophilia* with MICs ≤0.03 mg/L and four isolates with MICs 0.12, 0.25, 0.25 and 1 mg/L as described previously,^12^ mapping against GenBank reference LR134324.1. Clearly inactivating mutations (stop-gain and frameshift mutations) were identified and prioritized. Sequencing data from this project are available from the NCBI SRA (Accession PRJNA1417893).

## Results

### Antimicrobial susceptibility

Cefiderocol BMD performance assessment of 30 reference isolates yielded 100% essential and categorical agreement with *S. maltophilia* (*n* = 7), *Pseudomonas aeruginosa* (*n* = 10) and Enterobacterales (*n* = 8). All four reference *Acinetobacter* MICs were >1-log_2_-fold dilution higher than MICs performed for this study, including three very major errors. Attempts at discrepancy resolution with KB supported that these were particularly challenging isolates.

For the larger epidemiological survey of 162 isolates, a total of 60 *S. maltophilia*, 26 *P. aeruginosa*, 34 Enterobacterales and 42 other non-fermenting Gram-negative organisms were included. *Acinetobacter* (4) were included within the other non-fermenters, given low numbers in our population. This heterogeneous group of organisms included seven *Achromobacter xylosoxidans*, six *Chryseobacterium indologenes*, four *Elizabethkingia*, and six *Pandoraea* sp., among smaller numbers of other less common species. See Table S1 for complete organism distribution.

MIC distributions for the five reserve beta-lactams are shown in Table 1. Cefiderocol was the only agent with an MIC_90_ within the susceptible range across all organisms. The MIC_90_ for *S. maltophilia* was 0.25 mg/L (susceptibility breakpoint ≤1 mg/L), ≤4 mg/L for Enterobacterales (susceptibility breakpoint ≤4 mg/L), 8 mg/L for *P. aeruginosa* (susceptibility breakpoint ≤4 mg/L) and 16 mg/L for other non-fermenting GNRs (susceptibility breakpoint ≤4 mg/L for *Acinetobacter* species only). Aztreonam-avibactam, the only other agent resistant to hydrolysis by both serine and MBLs, maintained similar activity against *S. maltophilia* and Enterobacterales. For *P. aeruginosa* and other non-fermenting GNRs, the cefiderocol MIC_90_ was lower than for any other comparator. Extrapolated susceptibility results were consistent with the MIC_90_ trends (Table 1). The susceptibility of cefiderocol non-susceptible isolates to the other agents was heterogeneous (Table S2).

### Bacteraemia cohort

We identified 55 episodes of bacteraemia secondary to a CR-GNRs (Table S1). Thirty-day all-cause mortality was 29%. Many patients had malignancy and/or severe neutropenia (Table 2). After applying extrapolated susceptibility criteria, cefiderocol demonstrated the greatest *in vitro* activity (88.9% susceptible), followed by ceftazidime-avibactam (60.0%) and aztreonam-avibactam (50.9%). Seven (12.7%) cefiderocol non-susceptible isolates were identified and described in Table S3.

**Table 2.** Patients with bacteraemia and chart data (*n* = 55)

Demographic	Percent
Female	38.2%
Male	61.8%
Age	
20–29	9.1%
30–39	12.7%
40–49	12.7%
50–59	16.4%
60–69	25.5%
70–79	18.2%
80–89	1.8%
90–99	3.6%
30 day all-cause mortality	29.1%
Comorbidities	
Solid organ malignancy	20.0%
Solid organ transplant	12.7%
Haematopoetic malignancy	36.4%
Bone marrow transplant	21.8%
Cystic fibrosis	3.6%
Diabetes	20.0%
Heart failure	9.1%
Chronic kidney disease	14.5%
Neutropenia	40.4%
Severe neutropenia	34.0%
Extrapolated susceptibility of isolates*	
FDC	88.9%
ATM-AVI	50.9%
C/T	41.8%
CZA	60.0%
MVB	45.5%

^*^‘Extrapolated’ breakpoints applied: For CZA, C/T and FDC without formal interpretations, CLSI M100 35th Ed *P. aeruginosa* applied. For ATM-AVI without interpretations, FDA Enterobacterales breakpoints applied. For MVA, CLSI M100 35th Ed MEM applied for *P. aeruginosa*, *Acinetobacter*, *Stenotrophomonas*, other non-Enterobacterales or 33rd Ed MEM for *Burkholderia* spp.

### WGS of *S. maltophilia*

We used WGS to screen clinical isolates for disruptive mutations in genes that were previously reported to contribute to cefiderocol resistance. One isolate with a high MIC carried a stop-gain mutation in *mrcB* (encoding PBP1b, c.916C>T/p.Gln306*), while two others harboured a disruptive in-frame deletion in the same gene (c.2408_2410delCTG/p.Ala804del). The fourth isolate harboured a stop-gain mutation in *pbpC*, encoding PBP1c (c.1859C>A/p.Ser620*). None of the highly susceptible strains carried disruptive mutations in either of these PBP-encoding genes or in other annotated PBPs present in the *S. maltophilia* reference genome. While several high severity mutations were noted in genes affecting iron transport (*cirA*-family genes), such variants were not exclusive to isolates with higher MICs.

## Discussion

Relative to global surveillance projects such as SIDERO-WT^6^ and SENTRY^5^ for carbapenem non-susceptible isolates, our cefiderocol MIC_90_s were comparable for Enterobacterales (4 mg/L SIDERO-WT and SENTRY), higher for *P. aeruginosa* (8 mg/L versus 1 mg/L for SIDERO-WT) and slightly lower for *S. maltophilia* (0.125 versus 0.25 mg/L for SIDERO-WT and 0.5 mg/L for SENTRY). While the limited number of isolates examined here may partially explain these differences, they also highlight the importance of regional surveillance data derived from unique patient populations in characterizing expected resistance patterns.

Cefiderocol and aztreonam-avibactam were particularly active against *S. maltophilia* and other non-fermenting GNRs that are often implicated in cystic fibrosis and bone marrow transplant populations. The frequent avoidance of trimethoprim-sulfamethoxazole in the setting of haematologic malignancy due to marrow suppression underscores the importance of well-tolerated beta-lactam alternatives. Compared with SENTRY data, our aztreonam-avibactam MIC_50_ for *S. maltophilia* was 1-log_2_-dilution lower, with a comparable MIC_90_ suggesting efficacy in our local region.^13^

The optimal empiric therapy when infection with non-fermenting CR-GNRs is suspected is less clear. This uncertainty is amplified for less common genera like *Achromobacter*, *Chryseobacterium*, *Cupriavidus*, *Elizabethkingia*, *Pandoraea* and others for which both clinical data and interpretive breakpoints are limited.^2,14,15^  *Pandoraea* species, which harbour a chromosomal OXA-type beta-lactamase,^16^ were notable for four of five isolates having cefiderocol MICs >4 mg/mL. The pathogenicity of *Pandoraea* is unclear, however, and the organism is most frequently associated with cystic fibrosis and is often multidrug resistant.^17^ Importantly the isolation of two *Pandoraea* isolates from the bloodstream belies the allegedly reduced pathogenicity described by some authors.^17^

Since formal breakpoints remain unestablished for many of the organism–agent combinations considered here, we also calculated susceptibilities using ‘extrapolated breakpoints’ (Table 1). While such extrapolations cannot be used to infer clinical activity, they offer a pragmatic means for comparing *in vitro* activity across agents and organisms and reflect how susceptibility data are often interpreted clinically when only MICs are available. Reassuringly, our extrapolated susceptibility estimates generally aligned with the MIC_50_, MIC_90_ and established breakpoint interpretations (Table 1). While cefiderocol generally showed the highest *in vitro* activity, cefiderocol non-susceptible isolates exhibited variable susceptibility to the other agents tested, emphasizing both the heterogeneity in resistance mechanisms and distinctive antimicrobial spectra of the agents tested (Table S2, S3). Altogether, these data support broad reserve-agent testing when CR-GNR pathogens are isolated.

Although only a small number of clinical *S. maltophilia* isolates were examined, our WGS analysis identified disruptive mutations in *mrcB* and *pbpC* that were associated with higher MICs, suggesting that such mutations contribute to cefiderocol resistance. The *mrcB* (PBP1b) and *pbpC* (PBP1c) genes encode two of the seven annotated PBPs in *S. maltophilia*,^18^ and PBP mutations have been implicated as a mechanism of cefiderocol resistance,^19^ either directly or relating to PBP expression. Data in other species suggest that cefiderocol has the highest affinity for PBP3 (*ftsI*),^20^ but the loss of PBP3 function may be lethal in *S. maltophilia.*^18^ The relatively modest elevation in MICs seen in our isolates may reflect the lower affinity of cefiderocol for PBP1b and PBP1c relative to PBP3 in *S. maltophilia*, as well as the protective effect of such mutations.^19^ Studies involving larger numbers of clinical isolates will be necessary to verify and further explore this association.

Limitations of our study include the small number of isolates and a lack of isogenic mutants to verify WGS findings. Additionally, the clinical outcome analysis was descriptive and not designed to assess treatment efficacy. Despite these limitations, our data provide regionally relevant susceptibility data to inform antimicrobial selection when CR-GNR-related infections are suspected and provide further opportunities to explore resistance mechanisms.

## Supplementary Material

dlag105_Supplementary_Data

## Supplementary data

Tables S1 to S3 is available as Supplementary data at *JAC-AMR* Online.
